# Chemical Structure Representation Standardization Is Needed to Generalize Metabolite-Pathway Involvement Prediction Across KEGG, Reactome, and MetaCyc Knowledgebases

**DOI:** 10.3390/metabo16060357

**Published:** 2026-05-26

**Authors:** Erik D. Huckvale, Hunter N. B. Moseley

**Affiliations:** 1 Markey Cancer Center, University of Kentucky, Lexington, KY 40536, USA; edhu227@uky.edu; 2 Superfund Research Center, University of Kentucky, Lexington, KY 40536, USA; 3 Department of Toxicology and Cancer Biology, University of Kentucky, Lexington, KY 40536, USA; 4 Department of Molecular and Cellular Biochemistry, University of Kentucky, Lexington, KY 40536, USA; 5 Institute for Biomedical Informatics, University of Kentucky, Lexington, KY 40536, USA

**Keywords:** pathways, compounds, data curation, InChI canonicalization, machine learning, neural networks, model generalizability, KEGG, Reactome, MetaCyc

## Abstract

**Background/Objectives**: Due to the utility of knowing the pathway involvement of metabolites detected in biological experiments, knowledgebases such as the Kyoto Encyclopedia of Genes and Genomes (KEGG), Reactome, and MetaCyc have annotated compound entries to specific pathways defined by the knowledgebase. However, these compound-pathway annotations are largely incomplete and are costly to obtain experimentally or curate from published scientific literature. This metabolite-pathway annotation incompleteness problem is amenable to machine learning (ML)-based solutions. But to date, no machine learning model has been trained on all three knowledgebases to maximize its performance and robustness. This may be due to inconsistencies in chemical structure representation that can confuse a model and greatly reduce generalizability. **Methods**: We constructed a new training dataset with roughly 50,000,000 entries using compound-pathway annotations derived from KEGG, Reactome, and MetaCyc. We trained and tested a multitask classification, graph convolutional neural network-like model that classifies compound involvement with 8056 pathways that have unique pathway representations, based on annotated compound chemical structures represented with chemical substructure features. While the initial dataset contained inconsistencies in chemical structure representations across knowledgebases, we alleviated this issue by standardizing chemical structure representation using InChI (IUPAC International Chemical Identifier) canonicalization. We compared the performance of the non-standardized versus the standardized dataset and quantified their generalizability by comparing training set compounds to their knowledgebase cross-references. **Results**: While the non-standardized dataset scored a mean Matthews correlation coefficient (MCC) of 0.8725 ± 0.0064, the standardized dataset scored an MCC of 0.9036 ± 0.0033. When comparing model generalizability, the non-standardized chemical structure representations had a huge 0.2687 drop in mean MCC, while the standardized chemical structure representations only had a 0.0384 drop in mean MCC. **Conclusions**: We constructed the largest ML-ready dataset for predicting compound-pathway involvement to date. Next, we constructed, trained, and evaluated the highest performing ML model capable of predicting the highest number of pathway annotations to date. We discovered that standardizing chemical structure representation is an essential step when predicting novel chemical structures.

## 1. Introduction

Pathways are networks of interconnected chemical reactions and interacting biomacromolecules within cells and organisms. If a chemical compound is involved as a product, reactant, or other small-molecule participant in a chemical reaction, it is de facto associated with that reaction. If a particular reaction takes place in a “pathway”, the compounds associated with that reaction are considered to be associated with that “pathway” [1,2,3]. In this context, “pathway” can be a metabolic pathway, signaling pathway, biological process, disease process, or other biological concept with a graph-like representation of molecular interactions. When researchers detect various compounds in the biological samples of their experiments, it is highly useful to know which pathways the detected compounds are involved in, since such information provides insight into the biological functions of the compounds. This facilitates drug discovery, provides insight into the causes and treatment of disease, and aids biological research overall. Because of this interpretative utility, the pathway associations of compounds are annotated in knowledgebases such as the Kyoto Encyclopedia of Genes and Genomes (KEGG) [4,5,6], Reactome [7], and MetaCyc [8]. However, these knowledgebases are grossly (at least 50%) incomplete, as there are many compounds without any pathway annotations, and determining the pathway involvement experimentally is time-consuming and costly. A simple comparison of the compound and pathway entries in the three knowledgebases (see Table 1) clearly demonstrates that no single knowledgebase contains a majority of both compound and pathway entries. Likewise, the reactions in KEGG and MetaCyc represent a minority of known enzymatic reactions in other knowledgebases such as BRENDA: KEGG + MetaCyc represent roughly 20,000 reactions, compared to over 40,000 reactions in BRENDA, according to a comparison provided by MetAMDB [9]. At the compound level, KEGG and MetaCyc have roughly 9000 and 14,600 compounds, respectively, while BRENDA has over 111,000 compounds [9]. When enzyme promiscuity is considered, KEGG, MetaCyc, and Reactome, even together, likely represent a minority of compounds and reactions in cellular metabolism, and thus, the current definitions of pathways in these knowledgebases are incomplete. Given that the current human-defined pathways are incomplete, with the surrounding chemical reactions missing from their definitions, and considering that discovering the pathway involvement of metabolites is time-consuming and costly, the field of metabolomics has faced a persistent need to fill in the missing pathway annotations in an efficient and cost-effective manner, thereby expanding the pathway definitions to become more complete and more descriptive of (cellular) metabolism.

To increase the number of pathway annotations available for interpretation of experimentally measured metabolites, several prior studies have prototyped machine learning models that predict pathway involvement based on a compound’s chemical structure representation, with varying levels of performance. Attempts to predict the pathway involvement of compounds most notably began with the work of Hu et al., in which chemical interaction data were used to predict 11 level 2 metabolic pathway categories found in KEGG [10]. Building on the work of Hu et al., Baranwal et al. created a dataset representing compounds in SMILES format [11] along with their mappings to one or more of the 11 KEGG level 2 metabolic pathway categories. Baranwal et al. trained a multi-output graph neural network [12] with 11 outputs, one for each pathway category, where compounds were represented as graphs and information about their molecular structure was used to predict their pathway involvement. Yang et al. [13] and Du et al. [14] later proposed different variants of graph neural networks to predict these same pathway categories using the same dataset. Huckvale and Moseley discovered that the results of the models trained on this initial dataset were invalid [15] due to exact duplicates within the dataset, leading to data leakage and an overoptimistic estimate of model performance [16]. As a result, Baranwal et al. published a corrected version of the paper with the duplicate samples removed from the dataset [17]. All studies published before our 7 May 2024 publication [18] used either a multi-classifier or a set of binary classifiers implementing a one-vs.-rest classification approach [19] and only predicted 11 or 12 level 2 metabolic pathways defined in KEGG, which have very limited practical application, especially for pathway enrichment analyses. Since Baranwal et al. met the proper standards of scientific computational reproducibility by providing their code and data, we were able to train their model over 50 CV iterations and calculate MCC, resulting in a mean MCC of 0.7642 and a standard deviation of 0.0137 (Table S1), providing representative performance of models generated prior to 7 May 2024. Moreover, it is important to report model performance in MCC due to the high imbalance in the training and testing datasets. With high imbalance, MCC has an advantage over the F1 score, which ignores true negatives, and a major advantage over accuracy, which ignores false positives and false negatives within the numerator [20,21].

KEGG pathways are organized in a hierarchical fashion where there are seven top-level (level 1) pathway categories within which there are second-level pathway categories, and at the third level, we see individual pathways [22]. The 11 outputs of these past models were specifically predicting the second-level pathway categories under the ‘Metabolism’ top-level category. While these initial models were instrumental in demonstrating the ability to predict involvement based on information about a compound’s molecular structure, the reality is that there are far more pathways that are of biological interest. KEGG alone has over 500 pathways defined [22]. Meanwhile, Reactome and MetaCyc both have thousands of pathways defined [23,24]. Therefore, this is not a simple multi-output problem but rather an extreme classification problem [25,26] with thousands of different classes. One could train a multi-output model with thousands of outputs, but it is well known that as the number of classes increases while the dataset size remains the same, it becomes more challenging to accurately predict the increasing number of classes [27]. Alternatively, a separate binary classifier could be trained for each class, but in the case of pathway prediction, there are several small pathways with very few associated compounds. This results in many more negative entries than positive entries, and the severe class imbalance greatly reduces model performance [28].

Huckvale and Moseley resolved the extreme classification problem in metabolic pathway prediction by developing a multitask classification approach that cross-joins compound features with features representing a pathway, training a single binary classifier to predict whether the given compound is associated (i.e., involved) with the given pathway [18]. In this context, classifying a given compound as belonging to a given pathway represents a distinct classification task. With this technique, rather than the limited data set size (number of compounds only) needing to be shared amongst thousands of classes in a multi-output model, the dataset size increases, being multiplied by the number of classes (pathways), and only a single output is necessary. This is because, rather than a dataset entry being defined as a compound, it is defined as a compound–pathway pair. This reformulation of the metabolic pathway prediction problem demonstrated that a model can be trained in a computationally practical manner while predicting an indefinite number of pathways with sufficient performance. Firstly, Huckvale and Moseley demonstrated that not only can 12 level 2 metabolic pathways be effectively predicted (including the poorly performing pathway that everyone else left out) [18], but also that 172 level 3 pathways can be predicted [29]. This was followed by predicting all 502 pathways defined in KEGG using a dataset of 6485 compounds [30]. Going beyond KEGG, Huckvale and Moseley later demonstrated that models can effectively be trained to predict all 3985 Reactome pathways [31] and all 4055 MetaCyc pathways [32]. In addition, these studies demonstrated that training on all the pathways together with a single multitask classification model resulted in significant transfer learning across pathway-specific classification tasks, greatly improving pathway prediction compared to training a separate model for each pathway class in traditional one-vs-rest approaches.

**Table 1:** Description of the combined KEGG + Reactome + MetaCyc dataset compared to that of prior studies. “#” symbol normally represents “number of”.

Dataset	# Compound Features	# Pathway Features	# Unique Compounds	# Unique Pathways	# Entries	Reference
KEGG + Reactome + MetaCyc	34,474	27,208	16,640	8195	50,127,958	Current study
KEGG	16,509	11,321	6485	502	3,255,470	[30]
Reactome	6187	5386	1976	3985	7,874,360	[31]
MetaCyc	19,081	15,349	9847	4055	39,929,585	[32]

To handle a high number of pathways, Huckvale and Moseley entirely reformulated the problem to handle extreme classification by concatenating a compound feature vector with a pathway feature vector. The compound feature vector representation was made possible by the work of Jin et al. [33,34,35], who developed a graph-based atom coloring technique where the atoms of the compound are “colored” by the chemical substructure surrounding each atom. The atom coloring features for a compound are the counts of the atom colors (i.e., specific chemical subgraphs) present in the compound. This full enumeration of all chemical subgraphs of certain sizes present in each compound in a dataset creates an input neural network layer that is similar to the latent space produced by a graph convolutional neural network. Also, the resulting compound feature vectors can be viewed as feature vectors for chemical substructure tokens. The pathway features are likewise constructed by aggregating the compound features of the compounds associated with the pathway [18]. Multi-layer perceptron [36] layers are then trained using the combined compound-pathway feature vector as input. This approach is more practical than the previously used graph neural network methods, since many of the early (preprocessing) steps performed by graph neural networks have already been performed by atom coloring. Also, the introduction of pathway features, which cannot feasibly be represented as single definite graphs, prevents the direct use of most graph neural network methods.

With models being able to effectively predict the pathways annotated in these three major knowledgebases, an intuitive hypothesis is that the mean model performance and model robustness can be further improved by training a model on a dataset constructed from compounds and pathways in KEGG, Reactome, and MetaCyc combined. We will refer to this as the KEGG + Reactome + MetaCyc dataset. However, the challenge with combining knowledgebases is that their molfiles [37] have inconsistent chemical structure representations. This impacts both the way that the compounds are represented in compound features as well as the pathway features, which are derived from the compound features. We demonstrate in this work that these chemical structure representation inconsistencies confuse the model. By standardizing with InChI canonicalization [38,39,40], we make the chemical structure representations, and therefore the input features consistent, further improving the predictive performance of all pathways across all three knowledgebases. This is similar to the standardization methods used by PubChem; however, PubChem has different tautomeric preferences than InChI canonicalization [41]. We also demonstrate that the InChI-based standardization greatly improves the generalizability of the model, enabling better predictions of pathway involvement of novel chemical structure representations.

## 2. Materials and Methods

### 2.1. Creating the Initial (Non-Standardized) Dataset

To create the KEGG + Reactome + MetaCyc dataset, we downloaded compound molfiles from the KEGG, Reactome, and MetaCyc knowledgebases along with their pathway annotations. We used the kegg-pull Python package [42] to download KEGG data [30], and we downloaded Reactome [31] and MetaCyc [32] data directly using their respective web APIs. While the Reactome web API provides pathway definitions, its pathway annotations use ChEBI compound IDs, so we downloaded the Reactome molfiles from the ChEBI web API [43]. While KEGG, ChEBI, and MetaCyc had 18,673, 188,115, and 25,081 compounds documented, respectively, KEGG, Reactome, and MetaCyc had only 6584, 2061, and 10,032 compounds with pathway annotations specified, respectively (Table S2). Likewise, KEGG, Reactome, and MetaCyc had 522, 18,348, and 4240 pathways annotated (Table S2). This results in a total of 18,677 compounds and 23,110 pathways available across the three knowledgebases. However, not all documented compounds had valid molfiles, leaving 18,554 compounds (Table S3). The reduced number of compounds resulted in some pathways having no associated compounds. Since each pathway must have at least one annotated compound, this resulted in 22,504 pathways remaining (Table S3).

Using the md-harmonize Python package (v1.0.4) [35], we generated 0,1,2, and 3-bond atom colors for each compound from its molfile, creating 34,474 unique atom colors across the dataset after feature de-duplication. Exclusion of higher bond atom colors is based on the lack of new local chemical environment information provided by 4-bond atom colors [19]. This set of atom colors is used to generate the corresponding atom color feature vector for each compound, based on the counts of the atom colors present in the compound. The pathway feature vectors were the sums of the feature vectors of the compounds associated with each pathway. Thus, while the compound features are the counts of the atom colors present in each compound, the pathway features are the counts of the atom colors across all compounds within each pathway. Both the compound and pathway features were de-duplicated feature-wise and entry-wise, meaning that any duplicate features were removed, and any compound or pathway feature vectors with identical atom color counts were removed. Duplicate features are those that have the same value across all entries in the dataset and thus contain redundant information with respect to model training. Each group of duplicate pathway or compound feature vectors was combined to form a single entry. Since compound feature vectors potentially represent more than one compound, the pathway annotations of all compounds that they represent are combined. Both the compound and pathway features are additionally normalized using softmax entry-wise and min-max scaling feature-wise [18].

The pathway annotations served as the labels for the machine learning models, and we cross-joined the compound feature vectors with the pathway feature vectors, so that each entry in the dataset was a compound-pathway pair and the label was a binary value indicating whether the given compound is associated with the given pathway [18]. The cross-joined entries have no duplicates, preventing data leakage. However, since the compounds and pathways came from different knowledgebases, only the compounds and pathways from the same knowledgebase are cross-joined, creating a block diagonalization by knowledgebase in the final matrix of entries. This reduces the number of negative labels, since the compounds from one knowledgebase are necessarily not associated with the pathways of a different knowledgebase. However, de-duplication of compound and pathway entries can union entries from two different knowledgebases, so the block diagonalization overlaps with a few compound and pathway entries that span multiple knowledgebases.

Table 1 shows descriptive statistics of the dataset constructed from the combined data form KEGG, Reactome, and MetaCyc. There are more compound and pathway features, since both were generated from a larger statistical sample of molfiles. While one might expect that the number of compounds and pathways in the KEGG + Reactome + MetaCyc dataset would be the sum of the three knowledgebases, this is not the case due to the de-duplication of the feature vectors, indicating duplicate entries across the knowledgebases. The 16,640 unique compound feature vectors represent 18,554 compound entries across the knowledgebases. Likewise, 8195 unique pathway feature vectors represent 22,504 pathway entries across the knowledgebases (Table S3). However, a large number of completely duplicate pathways are present in Reactome, which is why the ratio of pathway entries to unique pathway feature vectors is above 2.5. Additionally, while past publications using the cross-join technique had a total number of entries equal to the number of unique compound feature vectors multiplied by the number of unique pathway feature vectors, since the dataset in this work did not pair compounds and pathways from different knowledgebases, the resulting total number of entries in the KEGG + Reactome + MetaCyc dataset was 50,127,958 post cross-join. The fraction of positive entries is 0.00543 (Table S3).

To evaluate model performance, we performed 100 random 90%:10% train/test splits, stratified across positive and negative entries in the full dataset [44]. For each train/test split, we created and trained a new model using only the 90% train set. We then tested this model on the 10% holdout test set, which was never used in model training. This cross-validation approach is a random jackknife analysis where 10% of the dataset is randomly left out for testing model performance. We performed 100 iterations to provide a reliable estimate of the model performance standard deviation. For each iteration, the training set duplicated its positive entries until the number of positive samples roughly equaled the number of negative entries. Specifically, we duplicated the positive entries by a number equal to the number of negative entries divided by the number of positive entries, rounded down. While this is not valid on the test set, upsampling the positive entries greatly helped with the negative predictive bias caused by class imbalance during training. The resulting train set was then used to train a multi-layer perceptron model, with hyperparameters tuned using the Optuna Python package (v4.0.0) [45]. The metric used for hyperparameter selection was the mean MCC of 24 train/test splits per hyperparameter trial (compared with 100 iterations for the final results). Table S4 specifies the hyperparameters selected as a result of the tuning. Once the model was trained, it was evaluated on the test set, collecting the number of true positives (TP), true negatives (TN), false positives (FP), and false negatives (FN). This enabled the calculation of metrics on each CV iteration, including the Matthews correlation coefficient (MCC) [20,21], F1 score, precision, recall, and accuracy. We counted the TP, TN, FP, and FN for all entries in the test set in order to calculate the overall mean, median, and standard deviation of MCC across the CV iterations. We also counted the same per entry, such that we could determine the per-knowledgebase MCC by counting the TP, TN, FP, and FN of entries belonging to each of the three knowledgebases.

To evaluate the performance when the model is trained on the entries of one knowledgebase and pathways are predicted for another knowledgebase, we split the KEGG + Reactome + MetaCyc dataset by knowledgebase, creating separate KEGG, Reactome, and MetaCyc dataset with the same input features. For each knowledgebase, we trained a model, using the same hyperparameters as in the CV analysis, on the compounds and pathways of that knowledgebase and used the resulting model to predict the pathways of the compounds in the other two knowledgebases. We calculated the MCC of the predictions for each combination of training knowledgebase and prediction knowledgebase.

### 2.2. Cross-Reference Analysis

To determine how well the model generalizes across different chemical structure representations from different knowledgebases, we determined how consistently the model predicted between pairs of compound entries cross-referenced between knowledgebases that represent the same molecule. We did this by identifying compound entries in one knowledgebase that had a cross-reference to a compound entry in another knowledgebase. The original compound entry and its cross-reference compound entry represent a cross-reference pair that has distinct molfile chemical representations in the two knowledgebases. We retrieved the cross-references between KEGG and MetaCyc, as well as KEGG and Reactome, using the kegg-pull Python package (v3.1.0) [42]. We retrieved the MetaCyc and Reactome cross-references using MetaCyc’s web API [8]. The three pairwise searches identified 9193 cross-reference pairs. Next, we trained the model on all entries in the dataset and used it to predict pathway associations for each molfile chemical representation in the cross-reference pair. This created two sets of pathway predictions, one for the compound entry from the first knowledgebase and the second for the compound entry from the second knowledgebase. We arranged the pairs so that the first compound in the pair was one of the compounds in the training set with known pathway annotations in the first knowledgebase, and the second compound was its cross-reference, which may or may not have known pathway annotations in the second knowledgebase. Using these 9193 pairs, we calculated the training set MCC based on the first knowledgebase pathway predictions for the first compound in each cross-reference pair and then calculated the cross-reference MCC based on the first knowledgebase pathway predictions of the second compound in the cross-reference pair. Thus, these MCC values are specific to the first knowledgebase pathways where the model training set included the first compound. This allows comparison of the training set and cross-reference MCC as a proxy for evaluating the overall generalizability of the model. Additionally, we counted how many cross-reference pairs have compound entries with identical counts of atoms of each color. This cross-reference analysis was performed on datasets with no standardization, InChI standardization, and atom and bond stereo atom coloring turned on and off.

### 2.3. Standardizing the Dataset

To create a standardized version of the KEGG + Reactome + MetaCyc dataset, we processed the molfiles using the obabel (Open Babel) command line tool (v3.1.1) [46]. This involved converting the molfiles to InChI format and then back into molfiles, which canonicalizes the chemical structure representation, i.e., selects a specific tautomeric/resonance form. We created the standardized dataset with the same detailed steps, but using the standardized molfiles. We also performed the CV, cross-knowledgebase evaluation, and cross-reference analysis detailed above. Hyperparameter tuning was also performed on the model trained on the standardized dataset, and Table S4 lists the selected hyperparameters. Standardizing the dataset resulted in fewer available molfiles, since not all molfiles were capable of being standardized properly, reducing the number of initial compounds from 18,554 to 18,527 in the standardized dataset. The further reduction in available compounds resulted in more pathways having zero associated compounds, reducing the number of initial pathways from 22,504 to 22,265 in the standardized dataset. Fewer compounds and pathways, as well as the standardization itself, resulted in fewer compound and pathway features and a lower number of unique compound and pathway feature vectors. Table S3 describes the impact of standardization on the number of unique pathways and compounds, as well as the number of features for both.

### 2.4. Experimenting with Atom and Bond Stereo

The md-harmonize package has an option to specify the atom or bond stereo in the atom colors [35]. Turning the atom or bond stereo off means that these details will not be included in the compound feature vectors and, therefore, the pathway feature vectors, which can impact model performance and generalizability. By default, both atom and bond stereo are turned on for the initial non-standardized dataset and the derived standardized dataset. To determine the impact of atom and bond stereo, we made three additional datasets derived from the standardized dataset: one with atom stereo turned on and bond stereo turned off, one with atom stereo turned off and bond stereo turned on, and one with both atom stereo and bond stereo turned off. We also performed the CV and cross-reference analyses for each of these three datasets. Hyperparameter tuning was additionally performed for these three datasets. In total, we trained five models, one for each dataset. Table S4 specifies the hyperparameters selected for the models resulting from each dataset. Different combinations of atom and bond stereo resulted in differing numbers of compound features and, therefore, pathway features, which in turn resulted in different numbers of unique compound and unique pathway feature vectors. Table S3 describes the impact of turning atom and bond stereo on and off on the number of features and feature vectors for the different datasets.

### 2.5. Hardware and Software Used

The hardware used for this work included compute nodes with up to 2 terabytes (TB) of random-access memory (RAM) and central processing units (CPUs) running at 3.8 gigahertz (GHz). The CPU was an ‘Intel (R) Xeon (R) Platinum 8480CL’. The CPUs were sourced from the Intel corporation in Santa Clara, CA, USA. The graphic processing unit (GPU) was an ‘NVIDIA H100 80 GB HBM3′ with 81.56 gigabytes (GB) of GPU RAM according to 1000 MB per GB definition. The GPUs were sourced from the Nvidia corporation in Santa Clara, CA, USA. Eight GPUs and eight cores were used to speed up hyperparameter tuning and CV analysis using multiprocessing, where a CV iteration was performed within one of eight processes at a time.

Table 2 details the computational resources used to train a model for each variant of the dataset. We observed maximal CPU and GPU utilization due to the efficient batching method developed by Huckvale and Moseley [30], which performs all batching GPU-side, compared to using multi-processing to perform the batching CPU-side, as is done with traditional deep learning batching methods. This 20-fold efficiency [30] is necessary for a dataset of this size, especially when combining all three knowledgebases together. In Table 2, we see that the non-standardized dataset took the most time to train and used the most RAM and GPU RAM.

All code for this work was written in version 3.10.12 of the Python programming language [47]. Data processing and storage were conducted using the Pandas (v2.2.3) [48], NumPy (v1.26.4) [49], and H5Py (v3.9.0) [50] packages. Models were constructed and trained using the PyTorch Lightning (v2.1.1) [51] package built upon the PyTorch (v2.3.1) [52] package, gradient descent performed using the Adam optimization algorithm [53]. The stratified train test splits were computed using the Sci-Kit Learn (v1.3.0) [54] package. Results were initially stored in an SQL database [55] using the DuckDB (v1.0.0) [56] package. Results were processed and visualized using Jupyter Notebooks (v1.1.1) [57], the Seaborn (v0.12.2) package [58] built upon the MatPlotLib (v3.7.2) [59] package, and the Tableau business intelligence application (v2024.3.3) [60]. Model training and testing were profiled for GPU and CPU utilization using the gpu_tracker package (v3.0.0) [61].

## 3. Results

### 3.1. Non-Standardized Dataset

#### 3.1.1. Model Performance

Table 3 presents the mean and median MCC from the CV analysis of models trained on datasets from the individual KEGG, Reactome, and MetaCyc knowledgebases compared to the KEGG + Reactome + MetaCyc dataset. As demonstrated by prior studies, KEGG by itself has comparable performance to MetaCyc, while Reactome significantly outperforms KEGG and MetaCyc. When training a model on all three knowledgebases, the overall mean and median MCC are between those of Reactome and the comparable MCC values of KEGG and MetaCyc. However, the standard deviation is roughly half that observed for the individual knowledgebases, indicating a large increase in model robustness.

Table 4 provides the mean and median MCC for predicting the pathways of each knowledgebase using a model trained on the corresponding knowledgebase alone, compared with a model trained on the KEGG + Reactome + MetaCyc dataset. When not standardizing the dataset, training on the compounds and pathways of all three knowledgebases increases the mean and median MCC when predicting the pathways in Reactome and MetaCyc and decreases their standard deviations as well. However, the mean and median MCC of KEGG pathways decrease, and their standard deviation increases, compared to training on the KEGG data alone, indicating increased confusion when predicting KEGG pathways.

Table 5 shows the mean MCC for training a model on the entries in one knowledgebase and predicting the pathways of the other two knowledgebases. Regardless of the combination, when training a model on the pathways of one knowledgebase and predicting the pathways of another knowledgebase, the mean MCC is very low. This indicates that while the model can generalize to novel compounds, it cannot generalize to novel pathway representations, making it very important that novel compounds predict the same pathways that the model was trained on.

#### 3.1.2. Cross-Reference Analysis

Table 6 shows the MCC when using the model trained on all entries to predict the pathways of the training set compounds compared with those of their cross-references, which may or may not have been in the training set. It also shows the number of cross-reference pairs that have identical atom color counts. There were 9193 compounds in the training set that had known cross-references, and out of those, only 1 had identical atom color counts. We see an MCC difference of 0.2687 when predicting on the cross-references, compared to the compounds in the training set.

### 3.2. Standardized Dataset

#### 3.2.1. Model Performance

Table 7 compares the mean and median MCC from the random jackknife cross-validation analysis of the standardized and non-standardized datasets. Standardizing the chemical structure representations prior to constructing the atom color features results in a slightly smaller dataset with 49,919,875 compound-pathway entries after feature and entry de-duplication, but a higher MCC and a much lower standard deviation.

**Figure 1:**
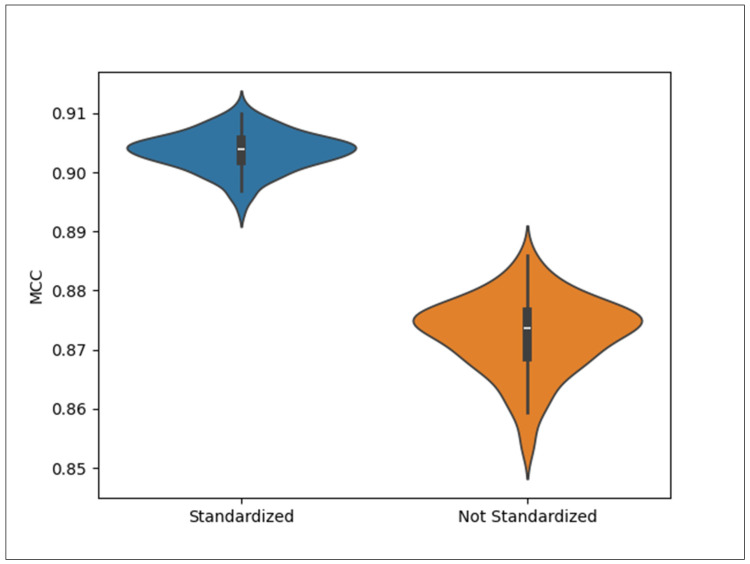
Violin plot of the distribution of the MCC of the standardized and non-standardized datasets across 100 CV iterations. A boxplot is included inside each kernel density plot.

Figure 1 provides the same results as Table 7, additionally displaying the distribution of the MCCs across the jackknife iterations. Both MCC distributions are unimodal, but the MCC distribution for the standardized dataset has a higher center, is less dispersed, and has less positive skew.

Table 8 compares the mean and median MCC of models trained on the datasets of the individual knowledgebases (which were not standardized) to those of the non-standardized KEGG + Reactome + MetaCyc dataset and its standardized version. The mean and median MCC for predicting the pathways of each knowledgebase are shown for each dataset. Training on the pathways of other knowledgebases increased the MCC and lowered the standard deviation for Reactome and MetaCyc pathways. Standardizing the dataset further increased the MCC and lowered the standard deviation for both. While KEGG pathways initially predicted more poorly when training on all three unstandardized knowledgebases as opposed to training on just KEGG, standardizing the dataset resulted in the MCC for KEGG pathways increasing and the standard deviation decreasing when training on other knowledgebases. However, this comparison cannot determine whether the improvement is due to standardization, the combined dataset, or both.

Table 9 contains the same results as Table 5 but for the standardized dataset. Standardizing the datasets for each knowledgebase prior to predicting on the pathways of another knowledgebase results in better performance than before, but the performance remains relatively low. Again, these results highlight how very important it is that the model is trained on the pathways to be predicted.

#### 3.2.2. Cross-Reference Analysis

Table 10 compares data and prediction consistency when the data are standardized and when they are not. The standardized dataset results in increased MCCs when predicting the compounds with known pathway annotations. More importantly, it greatly increases the MCC of the cross-references. Additionally, the difference between the training set compounds and their cross-reference compounds is much smaller, thereby significantly improving consistency. This is largely attributed to the increased number of cross-reference pairs that have identical atom color counts after standardizing, increasing from 1 out of 9193 to 7234 out of 9193 ≈ 78.7%.

### 3.3. Atom Stereo and Bond Stereo Inclusion

Table 11 shows the number of training set compounds with identical atom colors to their cross-references and how this number is affected by whether atom or bond stereo is turned on or off when generating atom colors. While standardizing resulted in many more compounds having identical atom colors, removing atom and bond stereo details from the atom colors led to even more compounds having identical colors, making the compound representation more consistent. See Table S5 for the same counts but for SMILES standardization instead of InChI. SMILES standardization resulted in less consistency, justifying the use of InChI for this case. The prediction consistency likewise increased, since we see a smaller MCC difference between the training set compounds and their cross-references. However, the best cross-reference MCC of 0.9239 was observed when using InChI standardization with both atom and bond stereo turned on.

Table 12 shows the results of the CV analysis of the four combinations of atom and bond stereo being turned on and off for the standardized dataset. While excluding atom and bond stereo details results in greater prediction and data consistency between cross-references, it reduces the overall model performance and robustness. See Table S6 for these results across all metrics in addition to MCC, and for all combinations of whether the data are standardized and whether atom or bond stereo areturned on or off. Again, the best performance was observed with atom and bond stereo on, producing a mean MCC of 0.9036 ± 0.0033. While the models’ precision was lower, ranging from 0.7975 to 0.8629, the recall was higher, ranging from 0.9473 to 0.9596. This resulted in the MCCs of all models being above 0.87 (Table S6).

## 4. Discussion

We combined the KEGG, Reactome, and MetaCyc knowledgebases together to create a single dataset comprising 13,902 unique compound feature vectors, 8056 unique pathway feature vectors, and 49,919,875 compound-pathway entries (Table S3). With the new combined dataset, the robustness of the resulting models improved to a mean MCC of 0.9036 ± 0.0033, with the standard deviation less than one-third of that reported in all prior published results. These are the best results published so far and are far better than older multi-classifiers or one-vs-rest binary classifiers; the best-performing multi-classifier has a mean MCC of 0.7642, and the best-performing one-vs-rest binary classifier has an average MCC of 0.7677 [19]. Moreover, all models prior to our May 7, 2024, publication [18] predicted only 11 or 12 level 2 KEGG metabolic pathways, compared with 22,265 pathways (8056 with unique representations) predicted by the extreme classification model presented here. The high level of performance presented here is due to four major innovations. One innovation is the cross-join of metabolite and pathway features, which allows the use of a single multitask classification model for this problem. The second innovation is the generation of metabolite atom coloring chemical subgraph features that can be combined to create pathway atom coloring features, which makes the cross-join possible. Also, the enumeration of all chemical subgraphs reproduces a latent space similar to what is generated from a graph convolutional neural network. The third innovation is the integration of the KEGG, MetaCyc, and Reactome knowledgebases using InChI canonicalization into a single large dataset with 49,919,875 entries, the largest dataset created for this purpose so far in the field. Do not forget that “Data is King!” in machine learning. The fourth innovation is the use of a custom data loader that performs the cross-join in GPU RAM, which speeds up model training by roughly 20-fold, making model training and hyperparameter tuning pragmatically possible.

The extreme classification model performance when predicting Reactome pathways and MetaCyc pathways additionally improved, indicating transfer learning across the knowledgebases. More precisely, the multitask classification approach demonstrates transfer learning between classification tasks, where Reactome pathway prediction represents one task and MetaCyc pathway prediction represents another task. However, KEGG pathway prediction performance decreased. The lower performance and robustness of KEGG pathways when trained along with the other two knowledgebases were caused by confusion introduced by inconsistent chemical structure representations between the knowledgebases. Prior to standardizing chemical structure representations, one might conclude that it was advisable to use a model trained on KEGG pathways only when predicting KEGG pathways. However, standardizing the chemical structure representations with InChI canonicalization evidently corrected and/or compensated for this discrepancy when training a model on all three knowledgebases, with KEGG pathway prediction performance improving. Therefore, we recommend training a single model to predict pathways from all three knowledgebases, as long as its training dataset was appropriately standardized. Chemical structure representation standardization further improved Reactome and MetaCyc pathway prediction performance as well. Also, the superior prediction performance for Reactome pathways versus KEGG and MetaCyc pathways implies that Reactome pathway definitions may be of high quality, compared to KEGG and MetaCyc. These results, taken together, indicate that standardizing the chemical structure representation of compounds significantly improves both model performance and robustness by enabling additional transfer learning between knowledgebase pathway classification tasks and/or preventing confusion, depending on one’s perspective.

Moreover, our cross-reference analyses demonstrated high inconsistency in chemical structure representations across knowledgebases with only 1 out of 9193 cross-reference pairs having identical atom coloring feature vectors. After chemical structure representation standardization, consistency across knowledgebases increased dramatically to 7234 out of 9193 ≈ 78.7%. By removing these inconsistencies in chemical structure representation, the drop in MCC for the cross-references decreased from 0.2687 without standardization to 0.0384 with standardization. Also, the standardized cross-reference MCC of 0.9239 represented the highest performance. Thus, the resulting models are more generalizable when predicting on compound entries outside of the training data while also maintaining high prediction performance. Therefore, it is essential to standardize the data prior to predicting metabolic pathway involvement. To our knowledge, investigations into data-engineering techniques to maximize model generalizability across different knowledgebases with different chemical structure representations have not been previously published.

Also, the method of standardization matters. Using SMILES for standardization was less useful than using InChI (Table S5). Also, the three knowledgebases have their own standardizations. However, different standardizations can have different tautomeric, resonance, and ionization preferences in chemical structure representation, which is illustrated by the poor performance when the three knowledgebases were combined without a separate standardization step. Likewise, PubChem’s standardization has a 60% inconsistency with InChI canonicalization [41]. Again, this all supports the use of a single chemical structure representation standardization method prior to training and predicting metabolic pathway involvement.

While the multitask classification models presented here have significantly higher performance than all prior published results, there are still limitations. While these models generalize to novel chemical structure representations, they do not generalize well to novel pathways. This is evident from the poor performance when building models trained on one knowledgebase and then predicting the pathways of another knowledgebase. When predicting metabolic pathways for novel compounds, we recommend predicting only pathways that the model was trained on. Further research is required to determine ways to generalize to novel pathways.

The addition of multi-layer perceptron (MLP) neural network layers complements the cross-join technique for extreme classification since input features in a graph format cannot be cross-joined with vectorized pathway features. If a graph2vec approach is used to vectorize the graph features prior to cross-joining with the pathway feature vectors, GPU memory limitations still arise for a dataset of this size, which we have directly tested. If the batch size is reduced to process a smaller number of compounds and prevent the graph neural network from outstripping GPU memory, it would be too small for the model to train in a reasonable amount of time. One could batch the compounds alone, perform graph2vec, and then cross-join with the pathway features, but current batching techniques, as provided by deep learning libraries such as PyTorch, are preformed on the CPU side with multiprocessing, where additional time is needed for transferring data between processes. However, we needed to create our own batching mechanisms performed entirely on the GPU side and in the same process, in order to practically train a model on a dataset of this size. Our custom batching method (data-loading method) improved GPU utilization by 20-fold, making the current model training, testing, and evaluation practical on a dataset with 49,919,875 entries. Special batching techniques would need to be developed to allow the use of a graph neural network followed by a cross-join of the vectorized compound representations. Such batching techniques are non-trivial to implement for graph data. However, here we demonstrate excellent performance using vector representations with atom color chemical subgraph features that input into MLP layers. Results may be improved if a batching technique with graph representations is implemented and efficiently performed on the GPU side, making the batching practical for a dataset of this size.

## 5. Conclusions

The KEGG + Reactome + MetaCyc dataset contains 13,902 uniquely represented compounds, 8056 uniquely represented pathways, and 49,919,875 compound-pathway entries. Our extreme classification MLP models can predict 22,265 pathways (8056 having unique representations) with a mean MCC of 0.9036 ± 0.0033, the best results published so far. As recommended, this training dataset was standardized with atom and bond stereo specified. The chemical structure representation standardization using InChI canonicalization significantly improves model performance and is essential for generalizability when predicting metabolic pathway involvement for novel compounds that would otherwise have an inconsistent chemical structure representation compared with the model. At this time, we recommend predicting the same pathways that the model was trained to predict. While there may be further improvement by using graph neural network methods, this requires custom batching techniques to be developed and implemented for training and testing to be practical on a dataset of this size (tens of millions of entries) without relying on multimillion-dollar GPU resources which are rare in academic settings. However, the multitask classification model presented here demonstrates excellent performance, robustness, and generalizability. It is a major step forward to provide additional pathway annotations for interpreting changes in metabolite experimental abundances, especially through pathway enrichment analysis.

## Figures and Tables

**Table 2:** Computational resource usage when training a model on each variant of the dataset.

Standardized	Atom Stereo	Bond Stereo	Resource	Unit	Amount
No	Yes	Yes	Compute time	Minutes	213.61
			CPU utilization	%	99.85
			GPU RAM	Gigabytes	30.35
			GPU utilization	%	98.13
			RAM	Gigabytes	3.53
Yes	No	No	Compute time	Minutes	114.25
			CPU utilization	%	99.64
			GPU RAM	Gigabytes	20.08
			GPU utilization	%	97.06
			RAM	Gigabytes	2.79
Yes	No	Yes	Compute time	Minutes	92.39
			CPU utilization	%	99.83
			GPU RAM	Gigabytes	18.12
			GPU utilization	%	96.73
			RAM	Gigabytes	2.70
Yes	Yes	No	Compute time	Minutes	57.12
			CPU utilization	%	99.36
			GPU RAM	Gigabytes	23.37
			GPU utilization	%	95.30
			RAM	Gigabytes	3.16
Yes	Yes	Yes	Compute time	Minutes	99.27
			CPU utilization	%	99.47
			GPU RAM	Gigabytes	24.48
			GPU utilization	%	96.37
			RAM	Gigabytes	2.69

**Table 3:** Overall MCC of the individual knowledgebases compared to the combined dataset.

Dataset	Mean MCC	Median MCC	Standard Deviation	Reference
KEGG + Reactome + MetaCyc	0.8725	0.8737	0.0064	Current study
KEGG	0.847	0.848	0.0098	[30]
Reactome	0.916	0.919	0.0149	[31]
MetaCyc	0.8446	0.8454	0.0101	[32]

**Table 4:** Per-knowledgebase performance when trained on each knowledgebase individually, compared to when trained on the combined dataset.

Training Dataset	Pathways’ Knowledgebase	Mean MCC	Median MCC	Standard Deviation	Reference
KEGG + Reactome + MetaCyc	KEGG	0.8138	0.8172	0.0175	Current study
KEGG Only	KEGG	0.847	0.848	0.0098	[30]
KEGG + Reactome + MetaCyc	Reactome	0.9221	0.9224	0.0048	Current study
Reactome Only	Reactome	0.916	0.919	0.0149	[31]
KEGG + Reactome + MetaCyc	MetaCyc	0.8548	0.8560	0.0092	Current study
MetaCyc Only	MetaCyc	0.8446	0.8454	0.0101	[32]

**Table 5:** Cross-knowledgebase evaluation for the non-standardized data.

Training Knowledgebase	Test Knowledgebase	Mean MCC
KEGG	Reactome	0.1196
KEGG	MetaCyc	0.0455
Reactome	KEGG	0.1996
Reactome	MetaCyc	0.1282
MetaCyc	KEGG	0.2312
MetaCyc	Reactome	0.2067

**Table 6:** Comparing the MCC of the compounds in the dataset to that of their cross-references. “#” symbol normally represents “number of”.

Training Set Compounds MCC	Cross-Reference MCC	MCC Difference	# Pairs with Identical Atom Colors
0.9129	0.6442	0.2687	1

**Table 7:** Comparing the mean and median MCC of the standardized dataset to the non-standardized dataset.

Standardized	Mean MCC	Median MCC	Standard Deviation
Yes	0.9036	0.9040	0.0033
No	0.8725	0.8737	0.0064

**Table 8:** Comparing the MCC when predicting pathways of each knowledgebase using a model trained on the individual knowledgebase, the non-standardized dataset, and the standardized dataset.

Training Dataset	Standardized	Pathways’ Knowledgebase	Mean MCC	Median MCC	Standard Deviation	Reference
KEGG + Reactome + MetaCyc	Yes	KEGG	0.8735	0.8735	0.0055	Current study
KEGG + Reactome + MetaCyc	No	KEGG	0.8138	0.8172	0.0175	Current study
KEGG Only	No	KEGG	0.847	0.848	0.0098	[30]
KEGG + Reactome + MetaCyc	Yes	Reactome	0.9428	0.9430	0.0040	Current study
KEGG + Reactome + MetaCyc	No	Reactome	0.9221	0.9224	0.0048	Current study
Reactome Only	No	Reactome	0.916	0.919	0.0149	[31]
KEGG + Reactome + MetaCyc	Yes	MetaCyc	0.8829	0.8833	0.0039	Current study
KEGG + Reactome + MetaCyc	No	MetaCyc	0.8548	0.8560	0.0092	Current study
MetaCyc Only	No	MetaCyc	0.8446	0.8454	0.0101	[32]

**Table 9:** Cross-knowledgebase evaluation for the standardized data.

Training Knowledgebase	Test Knowledgebase	Mean MCC
KEGG	Reactome	0.2440
KEGG	MetaCyc	0.1341
Reactome	KEGG	0.2777
Reactome	MetaCyc	0.2168
MetaCyc	KEGG	0.5117
MetaCyc	Reactome	0.3347

**Table 10:** Comparing the prediction and data consistency when standardizing and when not standardizing. “#” symbol normally represents “number of”.

Standardized	Training Set Compounds MCC	Cross-Reference MCC	MCC Difference	# Pairs with Identical Atom Colors
No	0.9129	0.6442	0.2687	1
Yes	0.9623	0.9239	0.0384	7234

**Table 11:** Comparison of prediction and data consistency for the four different combinations of atom and bond stereo inclusion. “#” symbol normally represents “number of”.

Standardized	Atom Stereo	Bond Stereo	Training Set Compounds MCC	Cross-Reference MCC	MCC Difference	# Pairs with Identical Atom Colors
No	On	On	0.9129	0.6442	0.2687	1
Yes	On	On	0.9623	0.9239	0.0384	7234
Yes	On	Off	0.8965	0.8679	0.0287	7465
Yes	Off	On	0.9320	0.9164	0.0155	8510
Yes	Off	Off	0.8871	0.8773	0.0098	8762

**Table 12:** CV analysis of the four combinations of atom and bond stereo inclusion.

Atom Stereo	Bond Stereo	Mean MCC	Median MCC	Standard Deviation
On	On	0.9036	0.9040	0.0033
On	Off	0.8707	0.8713	0.0063
Off	On	0.8985	0.8984	0.0047
Off	Off	0.8840	0.8859	0.0089

## Data Availability

All code and data for reproducing the results of this manuscript are available in the following Figshare items. Main manuscript results: https://doi.org/10.6084/m9.figshare.28701845; CV analysis of model and dataset of prior studies: https://doi.org/10.6084/m9.figshare.28701590.

## Supplementary Materials

The following supporting information can be downloaded at: https://www.mdpi.com/article/10.3390/metabo16060357/s1, Table S1: MCC of the dataset and model introduced by Baranwal et al. after 50 CV iterations, Table S2: The original number of compounds and pathways annotated in each knowledgebase, Table S3: Information on each dataset, Table S4: Hyperparameters selected by Optuna for each dataset, Table S5: Number of identical atom color counts between training set compounds and their cross-references when standardizing the data by converting to SMILES format, Table S6: CV analysis for all metrics and all combinations of standardization, atom stereo, and bond stereo.

## Abbreviations

The following abbreviations are used in this manuscript:

MCC	Matthews Correlation Coefficient
KEGG	Kyoto Encyclopedia of Genes and Genomes
TP	True Positives
TN	True Negatives
FP	False Positives
FN	False Negatives
TB	Terabytes
GB	Gigabytes
RAM	Random Access Memory
CPU	Central Processing Unit
GPU	Graphics Processing Unit
GHz	Gigahertz
MLP	Multi-layer Perceptron

## References

[1] Voet D., Voet J.G., Pratt C.W. (2016). Fundamentals of Biochemistry: Life at the Molecular.

[2] Berg J.M., Tymoczko J.L., Gatto G.J., Stryer L. (2019). Biochemistry.

[3] Nelson D.L., Cox M.M. (2021). Principles of Biochemistry.

[4] Kanehisa M., Goto S. (2000). KEGG: Kyoto encyclopedia of genes and genomes. Nucleic Acids Res..

[5] Kanehisa M. (2019). Toward understanding the origin and evolution of cellular organisms. Protein Sci..

[6] Kanehisa M., Furumichi M., Sato Y., Kawashima M., Ishiguro-Watanabe M. (2023). KEGG for taxonomy-based analysis of pathways and genomes. Nucleic Acids Res..

[7] Milacic M., Beavers D., Conley P., Gong C., Gillespie M., Griss J., Haw R., Jassal B., Matthews L., May B. (2024). The reactome pathway knowledgebase 2024. Nucleic Acids Res..

[8] Caspi R., Billington R., Keseler I.M., Kothari A., Krummenacker M., Midford P.E., Ong W.K., Paley S., Subhraveti P., Karp P.D. (2020). The MetaCyc database of metabolic pathways and enzymes—A 2019 update. Nucleic Acids Res..

[9] Starke C., Wegner A. (2022). Metamdb: Metabolic atom mapping database. Metabolites.

[10] Hu L.-L., Chen C., Huang T., Cai Y.-D., Chou K.-C. (2011). Predicting biological functions of compounds based on chemical-chemical interactions. PLoS ONE.

[11] Weininger D. (1988). SMILES, a chemical language and information system. 1. Introduction to methodology and encoding rules. J. Chem. Inf. Model..

[12] Asif N.A., Sarker Y., Chakrabortty R.K., Ryan M.J., Ahamed M.H., Saha D.K., Badal F.R., Das S.K., Ali M.F., Moyeen S.I. (2021). Graph Neural Network: A Comprehensive Review on Non-Euclidean Space. IEEE Access.

[13] Yang Z., Liu J., Wang Z., Wang Y., Feng J. (2020). Multi-Class Metabolic Pathway Prediction by Graph Attention-Based Deep Learning Method. 2020 IEEE International Conference on Bioinformatics and Biomedicine (BIBM).

[14] Du B.-X., Zhao P.-C., Zhu B., Yiu S.-M., Nyamabo A.K., Yu H., Shi J.-Y. (2022). MLGL-MP: A Multi-Label Graph Learning framework enhanced by pathway interdependence for Metabolic Pathway prediction. Bioinformatics.

[15] Huckvale E.D., Moseley H.N.B. (2024). A cautionary tale about properly vetting datasets used in supervised learning predicting metabolic pathway involvement. PLoS ONE.

[16] Yang C., Brower-Sinning R.A., Lewis G., KÄStner C. (2022). Data leakage in notebooks: Static detection and better processes. Proceedings of the 37th IEEE/ACM International Conference on Automated Software Engineering.

[17] Baranwal M., Magner A., Elvati P., Saldinger J., Violi A., Hero A.O. (2024). A deep learning architecture for metabolic pathway prediction. Bioinformatics.

[18] Huckvale E.D., Moseley H.N.B. (2024). Predicting the pathway involvement of metabolites based on combined metabolite and pathway features. Metabolites.

[19] Huckvale E.D., Powell C.D., Jin H., Moseley H.N.B. (2023). Benchmark dataset for training machine learning models to predict the pathway involvement of metabolites. Metabolites.

[20] Chicco D., Jurman G. (2020). The advantages of the Matthews correlation coefficient (MCC) over F1 score and accuracy in binary classification evaluation. BMC Genom..

[21] Cao C., Chicco D., Hoffman M.M. (2020). The MCC-F1 curve: A performance evaluation technique for binary classification. arXiv.

[22] KEGG Pathway Browser. https://www.genome.jp/kegg-bin/show_brite?br08901.keg.

[23] Reactome Pathway Browser. https://reactome.org/PathwayBrowser/.

[24] MetaCyc Pathway Browser. https://metacyc.org/META/class-tree?object=Pathways.

[25] Bengio S., Dembczynski K., Joachims T., Kloft M., Varma M. (2019). Extreme Classification (Dagstuhl Seminar 18291). Dagstuhl Rep..

[26] Varma M. (2019). Extreme classification. Commun. ACM.

[27] Moral P.D., Nowaczyk S., Pashami S. (2022). Why is multiclass classification hard?. IEEE Access.

[28] Guo X., Yin Y., Dong C., Yang G., Zhou G. (2008). On the class imbalance problem. 2008 Fourth International Conference on Natural Computation.

[29] Huckvale E.D., Moseley H.N.B. (2024). Predicting the Association of Metabolites with Both Pathway Categories and Individual Pathways. Metabolites.

[30] Huckvale E.D., Moseley H.N.B. (2024). Predicting the pathway involvement of all pathway and associated compound entries defined in the kyoto encyclopedia of genes and genomes. Metabolites.

[31] Huckvale E.D., Moseley H.N.B. (2025). Predicting the pathway involvement of compounds annotated in the reactome knowledgebase. Metabolites.

[32] Huckvale E.D., Moseley H.N.B. (2026). Predicting the pathway involvement of metabolites annotated in the MetaCyc knowledgebase. BMC Bioinform..

[33] Jin H., Mitchell J.M., Moseley H.N.B. (2020). Atom Identifiers Generated by a Neighborhood-Specific Graph Coloring Method Enable Compound Harmonization across Metabolic Databases. Metabolites.

[34] Jin H., Moseley H.N.B. (2021). Hierarchical Harmonization of Atom-Resolved Metabolic Reactions across Metabolic Databases. Metabolites.

[35] Jin H., Moseley H.N.B. (2023). md_harmonize: A Python Package for Atom-Level Harmonization of Public Metabolic Databases. Metabolites.

[36] Bisong E. (2019). The multilayer perceptron (MLP). Building Machine Learning and Deep Learning Models on Google Cloud Platform: A Comprehensive Guide for Beginners.

[37] Dalby A., Nourse J.G., Hounshell W.D., Gushurst A.K.I., Grier D.L., Leland B.A., Laufer J. (1992). Description of several chemical structure file formats used by computer programs developed at Molecular Design Limited. J. Chem. Inf. Model..

[38] Heller S., McNaught A., Stein S., Tchekhovskoi D., Pletnev I. (2013). InChI—The worldwide chemical structure identifier standard. J. Cheminform..

[39] Heller S.R., McNaught A., Pletnev I., Stein S., Tchekhovskoi D. (2015). Inchi, the IUPAC international chemical identifier. J. Cheminform..

[40] Goodman J.M., Pletnev I., Thiessen P., Bolton E., Heller S.R. (2021). InChI version 1.06: Now more than 99.99% reliable. J. Cheminform..

[41] Hähnke V.D., Kim S., Bolton E.E. (2018). PubChem chemical structure standardization. J. Cheminform..

[42] Huckvale E., Moseley H.N.B. (2023). kegg_pull: A software package for the RESTful access and pulling from the Kyoto Encyclopedia of Gene and Genomes. BMC Bioinform..

[43] Hastings J., Owen G., Dekker A., Ennis M., Kale N., Muthukrishnan V., Turner S., Swainston N., Mendes P., Steinbeck C. (2016). ChEBI in 2016: Improved services and an expanding collection of metabolites. Nucleic Acids Res..

[44] Verstraeten G., Van den Poel D. (2006). Using Predicted Outcome Stratified Sampling to Reduce the Variability in Predictive Performance of a One-Shot Train-and-Test Split for Individual Customer Predictions. Proceedings of the 6th Industrial Conference on Data Mining (ICDM).

[45] Akiba T., Sano S., Yanase T., Ohta T., Koyama M. (2019). Optuna: A Next-generation Hyperparameter Optimization Framework. Proceedings of the 25th ACM SIGKDD International Conference on Knowledge Discovery & Data Mining—KDD ’19.

[46] O’Boyle N.M., Banck M., James C.A., Morley C., Vandermeersch T., Hutchison G.R. (2011). Open Babel: An open chemical toolbox. J. Cheminform..

[47] Rossum G.V., Drake F.L. (2009). Python 3 Reference Manual.

[48] The Pandas Development Team Pandas-Dev/Pandas: Pandas 1.0.3. *Zenodo* 2020. https://zenodo.org/records/20127038.

[49] Harris C.R., Millman K.J., van der Walt S.J., Gommers R., Virtanen P., Cournapeau D., Wieser E., Taylor J., Berg S., Smith N.J. (2020). Array programming with NumPy. Nature.

[50] Collette A. (2013). Python and HDF5.

[51] Falcon W., Borovec J., Wälchli A., Eggert N., Schock J., Jordan J., Skafte N., Ir1dXD, Bereznyuk V., Harris E. PyTorchLightning/pytorch-lightning: 0.7.6 release. *Zenodo* 2020. https://zenodo.org/records/3828935.

[52] Paszke A., Gross S., Massa F., Lerer A., Bradbury J., Chanan G., Killeen T., Lin Z., Gimelshein N., Antiga L. (2019). PyTorch: An Imperative Style, High-Performance Deep Learning Library. arXiv.

[53] Kingma D.P., Ba J. (2014). Adam: A Method for Stochastic Optimization. arXiv.

[54] Pedregosa F., Varoquaux G., Gramfort A., Michel V., Thirion B., Grisel O., Blondel M., Müller A., Nothman J., Louppe G. (2012). Scikit-learn: Machine Learning in Python. arXiv.

[55] Chamberlin D., Liu L., Özsu M.T. (2009). SQL. Encyclopedia of Database Systems.

[56] Raasveldt M., Mühleisen H. (2019). Duckdb: An embeddable analytical database. Proceedings of the 2019 International Conference on Management of Data.

[57] Kluyver T., Ragan-Kelley B., Pérez F., Granger B., Bussonnier M., Frederic J., Kelley K., Hamrick J., Grout J., Corlay S., Loizides F., Scmidt B. (2016). Jupyter Notebooks—A publishing format for reproducible computational workflows. Positioning and Power in Academic Publishing: Players, Agents and Agendas.

[58] Waskom M. (2021). Seaborn: Statistical data visualization. J. Open Source Softw..

[59] Hunter J.D. (2007). Matplotlib: A 2D Graphics Environment. Comput. Sci. Eng..

[60] Salesforce (2024). Tableau Public.

[61] Huckvale E.D., Moseley H.N.B. (2024). gpu_tracker: Python package for tracking and profiling GPU utilization in both desktop and high-performance computing environments. arXiv.

