# Supplementary material for: Chemical Structure Representation Standardization Is Needed to Generalize Metabolite-Pathway Involvement Prediction Across KEGG, Reactome, and MetaCyc Knowledgebases
Source: Metabolites. 2026 May 26;16(6):357. doi: 10.3390/metabo16060357 (PMC13302955; doi:10.3390/metabo16060357)
Supplement: Supplementary file 1 [file metabolites-16-00357-s001.zip › metabolites-4311816-supplementary.pdf]

Supplemental Material

For

Chemical structure representation standardization needed to generalize metabolite-pathway involvement prediction across the Kyoto Encyclopedia of Genes and Genomes, Reactome, and MetaCyc knowledgebases

Erik D. Huckvale [1] and Hunter N. B. Moseley [1,2,3,4,5]*

[1] Markey Cancer Center, University of Kentucky, Lexington, KY, USA.
[2] Superfund Research Center, University of Kentucky, Lexington, KY, USA.
[3] Department of Toxicology and Cancer Biology, University of Kentucky, Lexington, KY, USA.
[4] Department of Molecular and Cellular Biochemistry, University of Kentucky, Lexington, KY, USA.
[5] Institute for Biomedical Informatics, University of Kentucky, Lexington, KY, USA.

*** Correspondence: hunter.moseley@uky.edu**

Table S1 – MCC of the dataset and model introduced by Baranwal et al after 50 CV iterations.

Mean MCC	Standard deviation
0.7642	0.0137

Table S2 - The original number of compounds and pathways annotated in each knowledgebase.

Knowledgebase	#Total Compounds	#Compounds With Pathway Annotations	#Pathways
KEGG	18673	6584	522
Reactome	188115	2061	18348
MetaCyc	25081	10032	4240

Table S3 - Information on each dataset.

Atom Stereo	Bond Stereo	Standardized	#Compounds	#Unique Compounds	#Compound Features	#Pathways	#Unique Pathways	#Pathway Features	#Entries	Proportion Positive
False	False	True	18527	12295	18208	22265	7987	15083	45671957	0.00549
False	True	True	18527	12531	19819	22265	7992	16384	46335783	0.00546
True	False	True	18527	13700	26220	22265	8051	21506	49373239	0.00531
True	True	False	18554	16640	34474	22504	8195	27208	50127958	0.00543
True	True	True	18527	13902	27718	22265	8056	22651	49919875	0.00528

Table S4 - Hyperparameters selected by Optuna for each dataset.

Atom Stereo	Bond Stereo	Standardized	Activation Function	Hidden Layer Size	Num Layers	Dropout	Learning Rate	Beta 1	Beta 2	EPS	Weight Decay	Prediction Threshold
False	False	True	ReLU	2173	4	0.055582	0.000792	0.445889	0.228346	0.000594	1.398695e-05	0.662897
False	True	True	ReLU6	1785	3	0.095894	0.000736	0.444590	0.563921	0.000419	9.891313e-07	0.623872
True	False	True	PReLU	1744	3	0.085695	0.000721	0.472054	0.485389	0.000980	6.524473e-05	0.624894

True	True	False	ReLU	1881	5	0.083652	0.000596	0.496627	0.247250	0.000799	6.812781e-05	0.649199
True	True	True	ReLU6	1229	3	0.063739	0.000719	0.565180	0.497934	0.000817	2.698780e-06	0.678975

Table S5 *- Number of identical atom color counts between training set compounds and their cross-references when standardizing the data by converting to SMILES format.*

	Bond Stereo On	Bond Stereo Off
**Atom Stereo On**	2066	2675
**Atom Stereo Off**	6031	7495

Table S6 - *CV analysis for all metrics and all combinations of standardization, atom stereo, and bond stereo.*

Atom Stereo	Bond Stereo	Standardized	Metric	Mean Score	Median Score	Standard Deviation
atom-stereo	bond-stereo	not-standardized	Accuracy	0.9984	0.9985	0.0001
atom-stereo	bond-stereo	not-standardized	F1 score	0.8696	0.8708	0.0070
atom-stereo	bond-stereo	not-standardized	MCC	0.8725	0.8737	0.0064
atom-stereo	bond-stereo	not-standardized	Precision	0.7975	0.7986	0.0129
atom-stereo	bond-stereo	not-standardized	Recall	0.9562	0.9566	0.0035
atom-stereo	bond-stereo	standardized	Accuracy	0.9989	0.9989	0.0000
atom-stereo	bond-stereo	standardized	F1 score	0.9031	0.9035	0.0036
atom-stereo	bond-stereo	standardized	MCC	0.9036	0.9040	0.0033
atom-stereo	bond-stereo	standardized	Precision	0.8629	0.8636	0.0092
atom-stereo	bond-stereo	standardized	Recall	0.9473	0.9476	0.0039
atom-stereo	no-bond-stereo	standardized	Accuracy	0.9984	0.9985	0.0001
atom-stereo	no-bond-stereo	standardized	F1 score	0.8675	0.8682	0.0070
atom-stereo	no-bond-stereo	standardized	MCC	0.8707	0.8713	0.0063
atom-stereo	no-bond-stereo	standardized	Precision	0.7929	0.7942	0.0139
atom-stereo	no-bond-stereo	standardized	Recall	0.9578	0.9580	0.0038
no-atom-stereo	bond-stereo	standardized	Accuracy	0.9988	0.9988	0.0001
no-atom-stereo	bond-stereo	standardized	F1 score	0.8974	0.8974	0.0051
no-atom-stereo	bond-stereo	standardized	MCC	0.8985	0.8984	0.0047
no-atom-stereo	bond-stereo	standardized	Precision	0.8467	0.8471	0.0120
no-atom-stereo	bond-stereo	standardized	Recall	0.9547	0.9547	0.0042
no-atom-stereo	no-bond-stereo	standardized	Accuracy	0.9986	0.9986	0.0001
no-atom-stereo	no-bond-stereo	standardized	F1 score	0.8817	0.8839	0.0099
no-atom-stereo	no-bond-stereo	standardized	MCC	0.8840	0.8859	0.0089
no-atom-stereo	no-bond-stereo	standardized	Precision	0.8159	0.8196	0.0202
no-atom-stereo	no-bond-stereo	standardized	Recall	0.9596	0.9603	0.0054
